# Soil amendments with ethylene precursor alleviate negative impacts of salinity on soil microbial properties and productivity

**DOI:** 10.1038/s41598-019-43305-4

**Published:** 2019-05-03

**Authors:** Hongwei Liu, Muhammad Yahya Khan, Lilia C. Carvalhais, Manuel Delgado-Baquerizo, Lijuan Yan, Mark Crawford, Paul G. Dennis, Brajesh Singh, Peer M. Schenk

**Affiliations:** 1 Plant-Microbe Interactions Laboratory, School of Agriculture and Food Sciences, The University of Queensland, Brisbane, Queensland 4072 Australia; 2 Hawkesbury Institute for the Environment, Western Sydney University, Penrith, New South Wales 2751 Australia; 3 Institute of Soil and Environmental Science, The University of Agriculture, Faisalabad, 38000 Pakistan; 4 The University of Agriculture Faisalabad, Sub-Campus Burewala, Vehari, 61100 Pakistan; 5 Centre for Horticultural Science, Queensland Alliance for Agriculture and Food Innovation, The University of Queensland, Dutton Park, Queensland 4102 Australia; 6 Cooperative Institute for Research in Environmental Sciences, University of Colorado, Boulder, CO 80309 USA; 7 Institute of Biodiversity, Friedrich Schiller University, Jena, 07749 Germany; 8 Department of Natural Resources and Mines, Toowoomba, QLD Australia; 9 School of Earth and Environmental Sciences, The University of Queensland, Brisbane, QLD 4072 Australia

**Keywords:** Microbial ecology, Soil microbiology, Applied microbiology

## Abstract

Some microbes enhance stress tolerance in plants by minimizing plant ethylene levels via degradation of its immediate precursor, 1-aminocyclopropane-1-carboxylate (ACC), in the rhizosphere. In return, ACC is used by these microbes as a source of nitrogen. This mutualistic relationship between plants and microbes may be used to promote soil properties in stressful environments. In this study, we tested the hypothesis that amendments of ACC in soils reshape the structure of soil microbiome and alleviate the negative impacts of salinity on soil properties. We treated non-saline and artificially-developed saline soils with ACC in different concentrations for 14 days. The structure of soil microbiome, soil microbial properties and productivity were examined. Our results revealed that microbial composition of bacteria, archaea and fungi in saline soils was affected by ACC amendments; whereas community composition in non-saline soils was not affected. The amendments of ACC could not fully counteract the negative effects of salinity on soil microbial activities and productivity, but increased the abundance of ACC deaminase-encoding gene (*acdS*), enhanced soil microbial respiration, enzymatic activity, nitrogen and carbon cycling potentials and *Arabidopsis* biomass in saline soils. Collectively, our study indicates that ACC amendments in soils could efficiently ameliorate salinity impacts on soil properties and plant biomass production.

## Introduction

Soil salinization is a prevalent phenomenon that affects approximately 20% of irrigated lands (227 Mha) worldwide^1^. The percentage of salt-affected lands is growing with time due to the improper management of land and water resources, such as the use of irrigation water with high salt level^2^. Recent estimates suggest that a staggering 50% of the world’s arable lands will be affected by salinity by the year 2050^3,4^. Saline soils are also one of the most challenging environments for plant production, and are often associated with disrupted biological, biochemical and hydrological cycles^5–7^. For instance, salinity adversely impacts soil carbon (C) and nitrogen (N) mineralization, respiration, residue decomposition, N cycling and bacterial and fungal growth rates, which are among the key factors leading to reduced fertility and productivity of saline soils globally^4,8–11^. Worldwide economic losses due to soil salinity are enormous. For example, the annual global income loss caused by salt‐induced losses in crop production on irrigated lands was estimated to be US$27.3 billion in 2013^12^. Given the importance of saline soils globally, identifying potential pathways to reduce plant stress is of paramount importance for improving agronomic productivity of salinity-affected soils and may help address food security issues over the following decades.

For nearly a century, organic and inorganic chemical amendments (e.g., using gypsum, farm manure)^12^, soil profile modification via tillage implementation^13^ and phytoremediation^14^ have been extensively used to reduce salt impacts on plant production. Such strategies have some important disadvantages such as (i) they require months to years to obtain expected outcomes; (ii) their efficacy in soil amelioration is highly variable and largely affected by soil edaphic properties; (iii) chemical amendments such as gypsum have become prohibitively costly in developing countries due to competition with industry materials^14^; and (iv) the use of phytoremediation in highly saline soils may be hindered because of poor plant growth^14^. In view of all the above, developing rapid and efficient alternative technologies for managing plant productivity in saline soils is urged.

Microbe-associated strategies to promote plant production in saline environments are increasingly more explored in recent decades, and could hold the key for improving crop production in such environments. Plant‐associated microorganisms in the rhizosphere, endosphere and phyllosphere play conjunctively important roles in plant health, development and functioning^15–17^ and have great potential to mitigate plant stress responses^18–20^. For instance, the degradation of 1-aminocyclopropane-1-carboxylate (ACC) by ACC-deaminase-producing microorganisms in the rhizosphere has been postulated as a promising mechanism that could be used to improve plant stress tolerance in saline environments. Salinity-stressed plants synthesize and accumulate large amounts of ACC in their tissues^4,21,22^. Part of the ACC is converted into ‘stress ethylene (ET)’ that inhibits plant growth by ACC oxidase^19,20^. In parallel, a significant amount of ACC is also released into the rhizosphere soil as root exudates^19^, which is utilized as a N and C sources by soil microbes that synthesize ACC-deaminase^20^. To maintain the equilibrium of ACC levels inside and outside the roots, plants may steadily release ACC into the soil^19,20^. This process ultimately results in a reduction of ET-associated plant stress linked to the presence of ACC-deaminase-producing microbes in the rhizosphere, which prevents conversion of ACC to ET. Efficient production of ACC deaminase is often employed as a key trait to assess microbes for their potential in alleviating various stresses such as drought, flood, cold as well as insect and pathogen attacks^17,23^.

Collectively, ACC is an important root exudate that plants can release into the rhizosphere and attract ACC deaminase-producers to favor plant growth and lower stress susceptibility. As such, amendments of ACC to soils using a series of different concentrations may hold a promising potential to ameliorate environmental stresses (e.g., salinity, drought and disease) on soil microbial properties and plant biomass production. However, empirical evidence for such a promising solution is lacking. Moreover, effects of ACC on soil microbial community composition and activities under different saline conditions remain unknown. To fill these knowledge gaps, we treated a control and an artificially-developed saline soil with ACC to investigate its impacts on the soil microbiome and productivity. Analysis of expression profiles of the *acdS* promoter region revealed that ACC concentrations (between 1–1,000 μM) induce ACC deaminase activity in bacteria^24^. We therefore chose to test different ACC concentrations (0, 50, 200 and 2,000 µM) around this range for their potential to affect related soil microbial properties, including the abundance of *acdS* genes. ACC in essence is a α-amino acid that is easily available for microorganisms and induce quick changes (within 10 days) in soil microbiome and properties^25^. Hence, due to the liability of amino acids in environments dominated by microbes, here we assessed short-term effects of ACC treatments on soil microbes and plant biomass production. We hypothesized that ACC amendments to soils: (i) affect the diversity and functions of soil microbial communities. The abundance of particular bacterial (e.g., *Microbacterium* and *Streptomyces*) and fungal ACC-deaminase producers are expected to be increased by ACC treatments^26^; (ii) improve microbial functions of saline soils, particularly ACC deaminase production potential due to increase of associated microbial populations and abundance of ACC-associated genes; and (iii) induce microbial functions that alleviate some of the negative effects of salinity on plant biomass production. We used a diverse range of culture-independent approaches to assess the responses of soil microbiomes to salinity and ACC treatments. We also used structural equation modelling (SEM) to gain a system-level knowledge on the importance of soil microbial community composition in regulating soil functions and plant production in response to ACC and salinity. Addressing the aforementioned knowledge gaps can lay the foundations for the development of effective agronomic practices aiming to minimize the effect of salinity on farm productivity.

## Results

### ACC amendments altered the composition of soil archaeal and bacterial communities and led to an increase in Actinobacteria in the saline soils only

**Figure 1:**
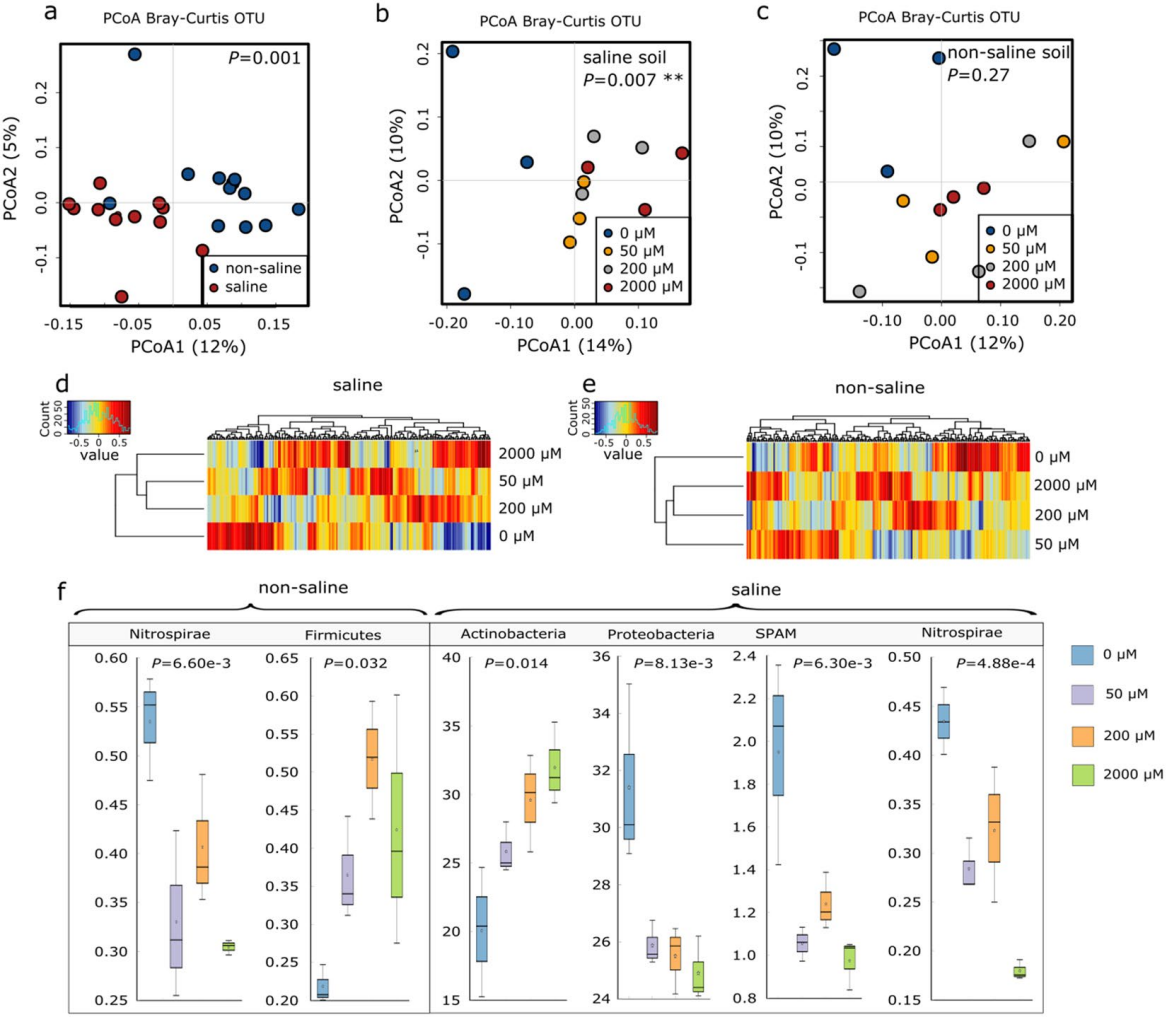
Effects of ACC and salinity treatments on the structure of soil prokaryotic (bacteria and archaea) community. Principal coordinates analysis **(**PCoA) summarizing variations in the microbial taxa based on 16S rRNA amplicon sequencing data (**a**–**c**); heatmap hierarchical clustering generated by using Spearman correlation coefficients of square root-transformed OTUs (**d**,**e**). Boxplots summarizing the relative abundances of bacterial phyla that were significantly influenced by ACC treatments. Boxplots indicate the first and third quartiles with the median value indicated by a horizontal line and the average was indicated by a star (**f**).

In general, our results from 16S rRNA amplicon sequencing indicated that soil archaeal and bacterial community composition were highly affected by ACC supply in saline soils, but not in non-saline soils, within the timeframe tested (Figs 1 and S1a). Principal Coordinate Analysis (PCoA) ordination, heatmap Spearman correlation and boxplots summarizing the ACC-induced changes in soil archaeal and bacterial community composition are shown in Fig. 1. As expected, salinity treatments caused a significant shift in soil archaeal and bacterial communities (P < 0.001, PERMANOVA, Fig. 1a). Importantly, the presence of salinity stress determined to which extend soil archaeal and bacterial community responded to ACC treatments. As such, the soil prokaryotic community composition was significantly influenced by the ACC treatments in the saline soils (*P* = 0.007, Fig. 1b and Table S1) while changes in the non-saline soils were minor (*P* = 0.27, Fig. 1c and Tables S2). Consistently, the resulting cluster heatmaps of square root-transformed OTUs using the Spearman correlation coefficients clearly differentiated ACC treatments in the saline soils, with the highest ACC concentration (2,000 µM) producing the largest impacts on soil microbiome (Fig. 1d). Salinity favoured higher relative abundances of *Actinobacteria*, *Armatimonadetes*, *CCM11b* and *Chlorobi* but decreased those of *Proteobacteria*, *Nitrospirae*, candidate division Spring Alpine Meadow (SPAM) and *Cyanobacteria* (Fig. S2). Further, salinity induced a less established network structure of the archaeal and bacterial community as a lower clustering coefficient was observed (Fig. S3 and Table S3).

At phylum level, the relative abundance of *Actinobacteria* increased with the ACC concentrations while *Proteobacteria*, *Nitrospirae* and SPAM decreased in the saline soils (Fig. 1f). When each of the dominant OTUs (>0.3% in proportion) was analyzed independently, we found that ACC treatments increased the relative abundance of an *Acidobacteria* (OTU_60523, *P* = 0.002) and a *Sinobacteraceae* (OTU33, *P* = 0.030) in the non-saline soils (Table S2). Whereas in the saline soils, ACC treatments led to increases of a *Microbacterium* (OTU_55567, *P* = 0.044) (Table S1). However, soil microbial alpha diversity, as indicated by the observed OTUs (richness), Simpson’s diversity index, predicated Chao1 and Shannon index, did not differ between different ACC or salinity treatments (Fig. S4).

### ACC treatments led to significant changes in fungal community composition in the saline soils

**Figure 2:**
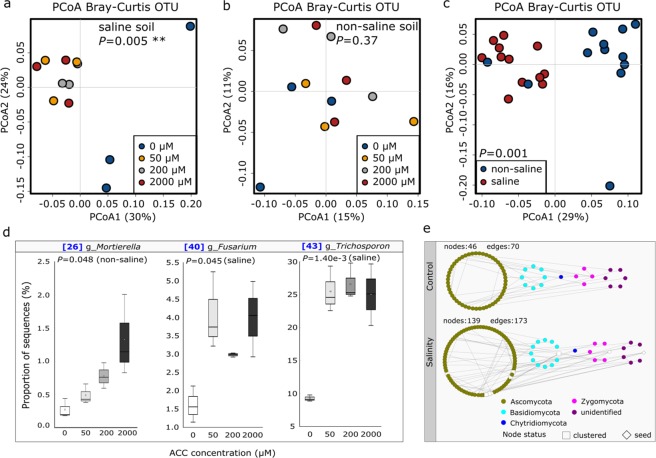
Principal coordinates analysis (PCoA) summarizing variations in soil fungal communities based on ITS amplicon sequencing data (**a**–**c**); soil fungal OTUs that were mostly affected by ACC treatments under saline and non-saline conditions (**d**). Boxplots indicate the first and third quartiles with the median value indicated by a horizontal line and the average indicated by a star. Changes in network structure of soil fungal communities in response to salinity treatments (**e**).

The soil fungal community, profiled by using ITS2 amplicon sequencing, was dominated by members of the *Ascomycota* (52.9%), *Basidiomycota* (34.5%), *Zygomycota* (5.8%) and an unidentified fungal group (6.3%). Similar to what we found for archaeal and bacterial community, fungal community composition was significantly affected by salinity treatments (*P* = 0.001, Fig. 2c). Furthermore, fugal community was significantly affected by ACC treatments in saline soils (*P* = 0.005, Fig. 2a), but not in non-saline soils (*P* = 0.37, Fig. 2b). When each of the dominant fungal OTUs (top 10) was analyzed independently, ACC treatments led to significant increases in relative abundance of a *Mortierella* (OTU26, *P* = 0.048) in the non-saline soils (Fig. 2d). Particularly, the yeast *Trichosporon* (OTU43, 8.9% in abundance) which was the dominant fungal population had its relative abundance increased by 2.8-fold (to 24.9%) in the saline soils (*P* = 1.40e-3) (Fig. 2d). In contrast to the bacterial network, the effect of salinity on the fungal network structure seems to be positive as higher clustering coefficient was obtained in the saline soils (Fig. 2e and Table S3). The saline soils were associated with a lower relative abundance of a *Pezizaceae* (OTU38) and higher abundances of two *Fusarium* populations (OTU39, 40), a *Haematonectria* (OTU41) and two members of *Trichosporon* (OTU43, 44) (Fig. S1b and Table S4).

### ACC treatments reduced the negative impacts of salinity on soil microbial enzymatic activity

**Figure 3:**
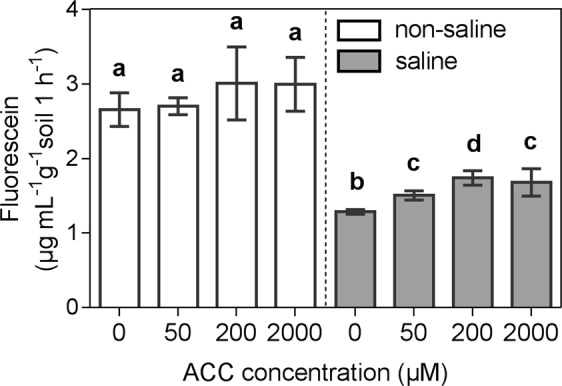
Total soil microbial enzymatic activity as indicated by FDA hydrolysis rates. Shown are mean values (n = 3) with SDs as error bars. Letters above columns indicate differences between treatments under saline and non-saline conditions (*P* < 0.05, one-way ANOVA, LSD).

Soil microbial enzyme activity (MEA), as indicated by the FDA assay, was reduced by salinity by 0.48 to 0.58 times (*P* < 0.001) (Fig. 3). Besides, MEA increased linearly with the ACC concentrations in the saline soils (R^2^ = 0.64, *P* = 0.0017), as opposed to non-saline soils, whose MEA was unaffected by ACC (Fig. 3).

### ACC treatments counteracted the negative impacts of salinity on soil C substrate utilisation

**Figure 4:**
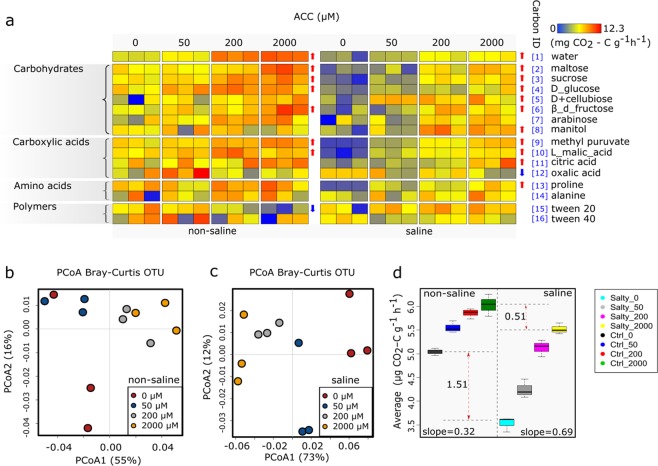
Heatmap (**a**) and principal coordinates analysis (PCoA) (**b**,**c**) summarizing variations in the substrate utilization profiles between samples based on carbon- utilisation (CO_2_ evolution) data. Each carbon source has a unique numeric identifier that is consistent between figures. Average microbial utilization of 15 C substrates in the saline and non-saline soils (**d**). The differences in soil C substrates utilization between the saline and non-saline soils became significantly smaller (by 33.8%) when the ACC concentrations increased from 0 to 2,000 μM.

Overall, the utilization of C substrates declined after salinity treatments in soils (*P* < 0.001), but this negative effect was significantly counteracted by all three levels of ACC amendments (*P* < 0.001) (Fig. 4). The rates of C substrate utilization significantly correlated with the ACC concentrations in both soils, and a significant interaction effect of ACC and salinity treatments was observed (*P* = 0.01) (Fig. 4b,c). The average C substrate utilization increased linearly with ACC concentrations in both the non-saline (R^2^ = 0.954, *P* < 0.001) and the saline soils (R^2^ = 0.980, *P* < 0.001) (Fig. 4d). After 2,000 µM ACC amendments, the average rate of C utilization was increased from 5.04 to 6.05 µg CO_2_-C g^−1^ h^−1^ (+20.04%) in the non-saline soils (Fig. 4d). The relevant change in the saline soils was larger, namely from 3.53 to 5.21 µg CO_2_-C g^−1^ h^−1^ (+56.37%) (Fig. 4d). When each C substrate was analyzed independently, soil basal respiration and utilization of ten C substrates (maltose, sucrose, D + glucose, D + cellubiose, β-d-fructose, mannitol, methyl pyruvate, L-malic acid, citric acid and proline) were stimulated by ACC treatments at three concentrations in the saline soils (Figs 4a and S5). Similarly, the basal soil respiration and utilization of six C substrates (maltose, sucrose, D + glucose, β-d-fructose, methyl pyruvate and malic acid) were stimulated by ACC treatments in the non-saline soils (Figs 4a and S5). In addition, tween 20 and oxalic acid were less metabolized after ACC treatments in the non-saline and saline soils, respectively. Lastly, nine C substrates (tween 40, proline, citric acid, L-malic acid, methyl pyruvate, β-d-fructose, D-glucose, sucrose and maltose) were utilized more rapidly in non-saline soils than saline soils (Figs 4a and S5).

### ACC treatments promoted genes related to soil C and N cycling and ACC deaminase synthesis in the saline soils only

**Figure 5:**
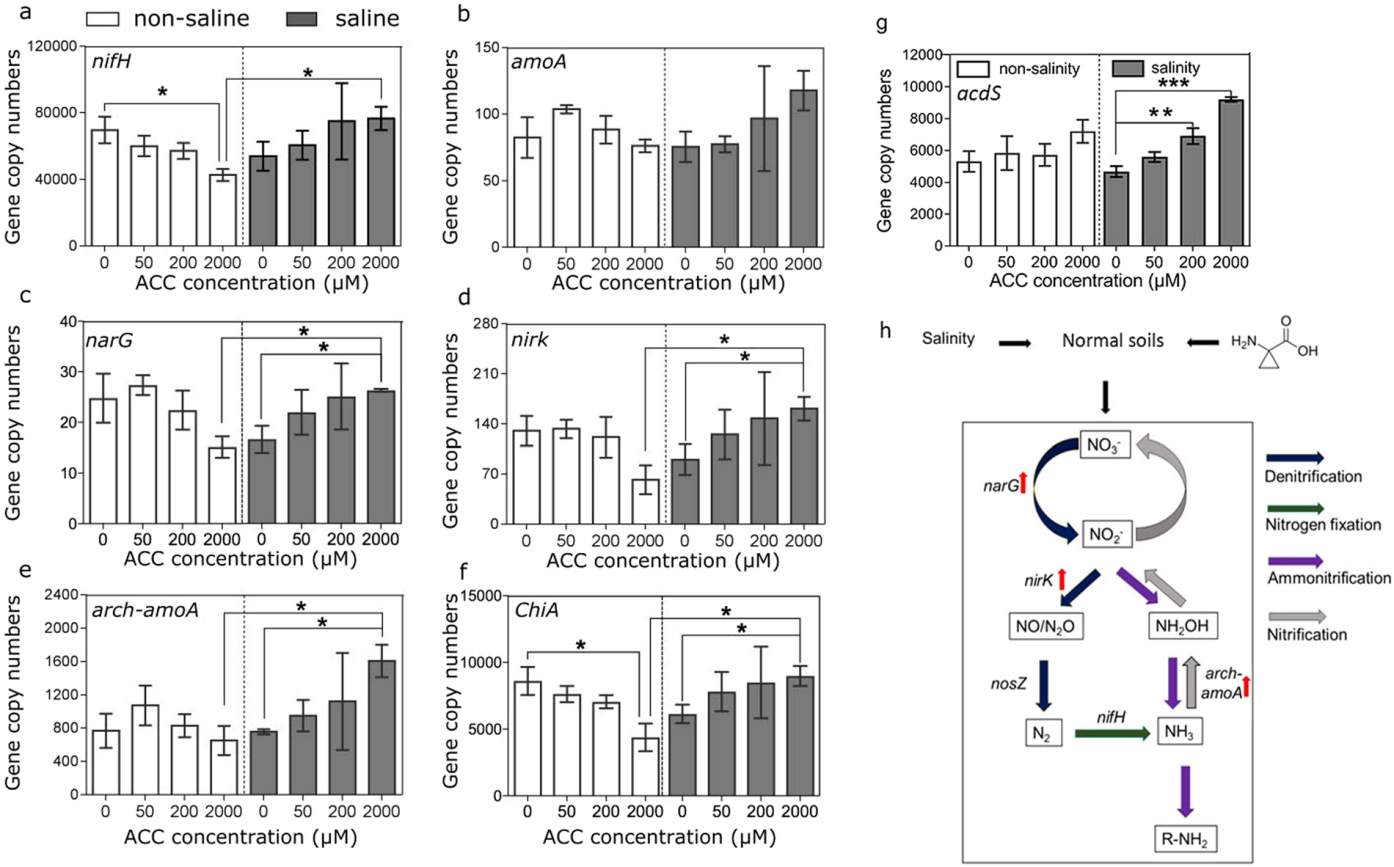
Effects of ACC treatments on copy numbers of *acdS* gene and soil nitrogen and carbon cycling potentials. The asterisks above columns indicate significant differences between treatments (*P* < 0.05, ANOVA, LSD) (**a**–**g**). Error bars are SEs (n = 3). Schematic flow diagram summarizes responses of nitrogen cycling genes to ACC treatments in the saline soils (**h**). Upward red arrows represent significant increases of *nirK*, *narG* and *arch-amoA* in copy numbers by 2,000 µM ACC treatments.

Microbial genes including *nifH*, *amoA*, *arch-amoA*, *nirK*, *narG* and *chiA*, which are known to be involved in key steps of soil C and N cycles, were measured to assess the effects of ACC on soil functional potentials in saline and non-saline soils. Overall, the effects of ACC on the N cycling genes were not statistically significant; however, there were significant interaction effects of ACC and salinity treatments for *nifH* (*P* = 0.01, ANOVA, Fig. 5a), *narG* (*P* = 0.01, Fig. 5c), *nirK* (*P* = 0.02, Fig. 5d) and *arch-amoA* (*P* = 0.047, Fig. 5e). Further analyses revealed that these interactions were driven by a significant decrease of *nifH* in the non-saline soils and significant increases of *narG*, *nirK* and *arch-amoA* in the saline soils by 2,000 µM ACC treatments (Fig. 5). In contrast, the abundance of *chiA* was significantly influenced by ACC treatments, and positively correlated to ACC concentrations in saline soils (Fig. 5f). Importantly, ACC treatments of 200 µM and 2,000 µM significantly increased the abundance of ACC deaminase-encoding gene *acdS* in saline soils by 1.48 (*P* = 0.001) and 1.97 (*P* < 0.001) folds, respectively (Fig. 5g). But this effect in non-saline soils was not significant (Fig. 5g). Overall, ACC treatments of 200 µM and 2,000 µM induced *acdS* gene in saline soils while abundances of certain C and N cycling genes were only increased by 2,000 µM ACC (Fig. 5h).

### ACC treatments improved the productivity of saline soils but could exert negative effects in non-saline soils when applied at high concentrations

**Figure 6:**
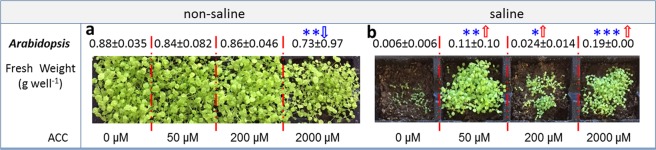
Plant biomass production of wild-type *Arabidopsis thaliana* (Col-0) in the saline and non-saline soils. The plant biomass production was recorded by measuring the fresh weight 2 weeks after sowing. In the non-saline soils, ACC amendments at 50 and 200 µM exerted no effects but 2,000 µM ACC reduced the plant biomass production (**a**); ACC amendments at all three levels (50, 200, 2,000 µM) alleviated detrimental effects of salinity on *A*.*thaliana* biomass (**b**). Stars in (**a**,**b**) represent significant differences between different ACC treatments (*P < 0.05, **P < 0.01, ***P < 0.001, ANOVA, Least Significant Difference (LSD)). Shown are mean values (n = 4) with SE as errors.

Salinity treatments severely hindered the biomass production of *A*. *thaliana* (Col-0) (−99.3%) within a two weeks’ cultivation (Fig. 6a,b). In non-saline soils, ACC at 50 and 200 µM did not influence the biomass of *Arabidopsis*, but 2,000 µM ACC was detrimental to biomass production (−17.3%) (Fig. 6a). In contrast, saline soils that received ACC treatments at 50, 200 and 2,000 µM were associated with significant improvements in the biomass of *Arabidopsis* relative to the 0 µM ACC control (4~32 folds) (Fig. 6b).

### SEM revealed that indirect beneficial effects of ACC treatments on soil functions and productivity were associated with changes in bacterial community structure

SEM explained a large proportion of the variation in soil function (78%) and plant biomass (97%) (Fig. S6). Salinity had multiple direct and indirect negative effects on plant biomass and soil functions (glucose utilization and MEA). Importantly, SEM provided evidence to suggest that microbial communities indirectly drove the plant physiological responses to ACC via changes in microbial activity (e.g. seed germination and/or plant growth). The correlation between the axes from our NMDSs and single taxa are available in Table S5.

**Figure 7:**
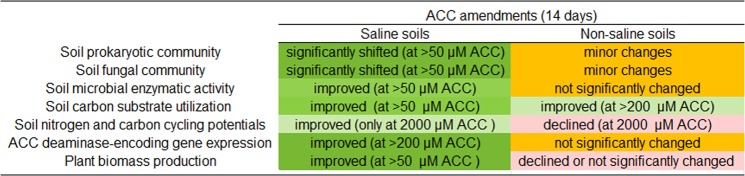
A table summarizing the effects of ACC amendments on soil microbial quality and plant productivity in the saline and non-saline soils. Green colours represent beneficial effects; the orange and pink represent minor changes and deleterious effects, respectively.

Lastly, overall effects of ACC amendments on soil microbial properties and productivity were summarized in Fig. 7, which indicates that ACC amendments provide more benefits to saline than non-saline soils.

## Discussion

Reduction of plant endogenous ET by the action of microbe-derived ACC deaminases may be among the key strategies that plants have developed to deal with stresses while coevolving with soil microorganisms^17–20^. In this study, we provided evidence to support that amendments of ACC to saline soils (EC_1:5_ = 8) can effectively (i) change the composition of soil bacterial, archaeal and fungal communities, (ii) improve multiple aspects of soil microbial functions including soil microbial enzymatic and metabolic activities as well as C and N cycling potentials, (iii) induce higher abundance of an ACC deaminase-encoding gene (*acdS*), and (iv) promote plant biomass production. These benefits were especially noticeable when a high concentration of ACC (2,000 µM) was used as a soil amendment. In contrast, we observed that amending ACC to non-saline soils had small or even deleterious effects on soil microbial properties and plant biomass production. The above findings provide new insights to the understanding of plant-microbe interactions in saline soils and may be used in the development of novel agronomic practices aiming to reduce negative impacts of salinity on soil properties and agronomic productivity.

### ACC amendments exerted a stronger effect on the microbiome of saline soils

The supplemented ACC may have provided soil microorganisms with additional N and C. Our findings suggest that ACC amendments alter microbial community composition in saline soils, but not in non-saline soils. Physiological stresses induced by salinity in soil microbes alter rates of microbial activity, which may determine to what extent they will be influenced by ACC amendments^5,6^. The fact that Proteobacteria, Nitrospirae and Cyanobacteria are Gram-negative and highly susceptible to salinity-induced osmotic stress may be associated with the reduced abundances of these groups in the saline soil microbiome^27^. ACC amendments in this study resulted in differential microbial phyla responses in saline and non-saline soils. In saline soils, increases in Actinobacteria correlated with increases in ACC levels in soils. Actinobacteria are usually abundant in soils and are essential to the global C cycles as they are involved in breaking down plant biomass^28^. This bacterial group, along with a wide range of bacterial and fungal species, possesses the *acdS* gene, which regulates the production of ACC deaminase^19,20,24^. A pronounced enrichment of Actinobacteria has been previously found in the rhizosphere (+2.3-fold) and endosphere (+3.1-fold) of grass species afflicted by drought^29^. This suggests plants under drought stress may induce an increase in abundance of members of that Actinobacteria. Similarly to salinity, drought stress also leads to the production of stress-ET^30,31^ and therefore the same strategy of decreasing endogenous plant ET levels via enhancing microbial ACC degradation may have been adopted. Furthermore, a *Microbacterium* had increased relative abundance with increasing ACC levels in saline soils. Many *Microbacterium* species/strains are ACC deaminase-producers that can improve plant growth and tolerance to abiotic stresses^32,33^. *Microbacterium* species have also been documented to be critical components for the bacterial consortium formed in the rhizosphere of *A*. *thaliana* that was infected with *Hyaloperonospora arabidopsidis*, the causative pathogen of downy mildew disease^34^.

Moreover, we observed that several groups of *Acidobacteria* displayed lower relative abundances after ACC amendments in saline soils. *Acidobacteria* comprise about 20% of bacterial communities in soils, and are highly phylogenetically diverse and active, but are rarely cultured^35,36^. *Acidobacteria* are often considered slow-growing oligotrophs and adopt a K-selected life strategy^37^. In the case of our study, an additional input of soil C and N after the microbially-mediated ACC degradations may have contributed to the decline of *Acidobacteria* by favoring fast-growing microbes^37^.

Within fungal communities, *Trichosporon* is a genus of anamorphic fungi with all species being yeasts. Interestingly, they are the most dominant fungal group in ACC-treated saline soils (24.9%). Despite yeast species having been less investigated for their interactions with plants, previous evidence points that they have plant growth promoting potentials^38^. For instance, inoculation of sugar beet roots with *Trichosporon asahii* significantly increased the plant growth and alleviated symptoms of *Rhizoctonia solani* infections^39^. Future experiments are recommended to isolate the fungus and investigate its capacity in producing ACC deaminases. Altogether, changes in soil archaeal, bacterial and fungal communities induced by ACC amendments were much more pronounced in saline soils than non-saline soils. This suggests that ACC plays a role in alleviating the osmotic stress caused by high salinity. Furthermore, the ACC-induced shifts in soil microbial structure may result in an alteration of soil functioning and thereby an effect on plant growth. It is worth pointing out that the observed increases in relative abundance of particular bacterial and fungal taxa may also be a result of microbial interactions. For example, *Chryseobacterium* species have been shown to concur with rhizobium^40^.

### ACC treatments mitigated the salinity-induced loss on soil microbial functions

Soil microbial activity plays a major role in organic matter decomposition and soil energy flow^41^. As such, any changes in soil microbial enzymatic and metabolic activities may consequently result in changes of plant growth due to modifications of available nutrients in the soil. Salinity-derived osmotic stress and ion toxicity (e.g., Na^+^) are known to exert damaging effects on microbial growth and activity^4^. Expectedly, we observed strong negative effects of salinity on soil microbial activities, which, however, were considerably counteracted by ACC amendments at all three concentrations tested. We demonstrated that the three tested levels of supplemented ACC increased the microbial enzymatic activity in saline soils, but not in non-saline soils. This is consistent with our findings that after ACC amendments archaeal, bacterial and fungal communities were significantly altered in composition in saline soils but mostly remained the same in non-saline soils. In addition, priming effects of ACC amendments on soil basal and C substrate-induced respiration were observed for both the non-saline and saline soils, but the effects on the latter were far more pronounced. Collectively, our findings suggest that overall microbial activities in saline soils are enhanced after ACC amendments, which may support plant growth and consequently biomass production.

### Implications of ACC amendments on soil N and C cycling potentials

Given that ACC is degraded into ammonia (NH_3_) and ketobutyrate (C_4_H_6_O_3_) and further utilized as N and C sources by soil microbes^19^, we investigated the impact of ACC amendments on soil N and C cycling potentials. Bacterial Chitinase group A encoded by *chiA* is produced by a variety of bacteria, which endows these microbes the capability to degrade environmental chitin^42,43^. The observed increase of *chiA* in the saline soils by ACC treatments correlated to the corresponding increases of Actinobacteria in relative abundance, which suggests that members of this phylum may be the source of this enzyme (e.g., *Streptomyces* sp.)^44^. The microbe-mediated biogeochemical cycles of N comprise N fixation, ammonification, nitrification and denitrification, through which N is converted into various chemical forms^45^. N fixation converts atmospheric N_2_ into biologically available ammonium (NH_4_^+^) by free-living and symbiotic N-fixing bacteria^46^. The gene *nifH* encodes a subunit enzyme of nitrogenase that catalyzes this process^47^. The decrease of *nifH* in the ACC-treated non-saline soils may have occurred because ACC provided microbes with extra N, leading to a suppression of N-fixing microbes. Nitrification is the biological oxidation of ammonia (NH_3_) to nitrate (NO_3_^−^) with nitrite (NO_2_^−^) as an intermediate. This process is driven by specific groups of ammonia-oxidizing archaea and ammonia-oxidizing bacteria using ammonia monooxygenase encoded by *amoA*^48,49^. Thus, there is indication of an enhanced nitrification in the ACC-treated saline soils driven by ammonia-oxidizing archaea. This may be a result of an additional supply of ammonia generated by the degradation of ACC in soils. Nevertheless, a cytochrome cd1 enzyme encoded by a Cu-containing enzyme encoded by *nirK* perform the stepwise reduction of nitrate (NO_3_^−^) or nitrite (NO_2_^−^) into nitrogen gas (N_2_), returning N to the atmosphere^50^. Increases in the abundances of most genes in N cycling and therefore in N turnover when ACC was supplemented to saline soils, indicate that soil microbial activities were generally stimulated in saline soils.

### Implications of ACC treatments on the production of ACC deaminase in soils

Our results suggest that ACC treatments lead to an increase in *acdS* abundance starting from the concentration of 200 µM. Even higher increases were revealed in soils exposed to 2,000 µM. This is consistent with previous findings that ACC deaminase production were enhanced by high ACC concentrations (1~2,000 µM) in some bacterial species (e.g. *Burkholderia*)^24^. The extent to which ACC deaminase was induced by the ACC treatments was not measured in our study, and this warrants future investigations. Our gene expression data indicate that ACC deaminase production is increased by high concentrations of ACC amendments in saline soils only, and even high level of ACC (200 and 2,000 µM) may not enhance the enzyme production in non-saline soils. This is consistent with other soil biological processes (e.g. C and N mineralisation), where salinity influenced their responses to ACC treatments.

### Implications of ACC treatments on soil productivity in both saline and non-saline soils

ACC amendments in saline soils significantly increased biomass production of the *Arabidopsis*, especially at a high concentration (2,000 µM). Such an enhanced biomass production in saline soils could be explained by two mechanisms that may have worked synergistically. Firstly, ACC amendments have potentially increased the ACC deaminase contents in soils (evidenced by the induced expression of *acdS* gene in this study), which degraded ACC released by salinity-affected plants and consequently lowered stress-ET levels in *Arabidopsis*. Such a mechanism may have enhanced *Arabidopsis* tolerance to salinity stress^18^. Secondly, soil fertility may have been improved as ACC represented an additional source of C and N after microbial degradation and eventually became available for plant uptake, culminating in *Arabidopsis* growth. The latter is supported by our SEM analysis (Fig. S6), which suggests that at least some of the effects of ACC on plant biomass are indirectly driven by changes in microbial community structure and functions. This result was maintained even after accounting for salinity in the model. Altogether, the above evidence supports our hypothesis that ACC promotes plant biomass production under stress conditions through the regulation of soil attributes.

Despite not tested, ACC may still remain in soils after two weeks’ incubation especially when used in high concentrations. If ACC remained, it could potentially enhance ET levels in *Arabidopsis*. There is conflicting evidence in the literature on whether ET negatively or positively alters plant physiology under saline stress^51^. While some studies demonstrate ET can enhance plant salinity tolerance (e.g., by activating the EIN3 (ETHYLENE INSENSITIVE 3) and EIL1 (EIN3-LIKE 1) transcriptional preventing reactive oxygen species accumulation)^52^, others provided evidence that ET inhibits root growth and elongation^53^. Therefore, the mechanisms through which ACC influences plant biomass production remains to be deciphered.

Overall, our results provide the foundation knowledge for the development of effective approaches to ameliorate salinity stress on soil properties and productivity. Despite we acknowledge that ACC amendment is not the most economically appealing, it provides an alternative to rapidly enhance the productivity of saline soils when cheaper approaches fail to be effective^13^. We would like to highlight that this approach could lead to negative impacts on plant biomass at high concentrations (2,000 µM) in non-saline soils. Similarly, ET can play an opposite role in other plant species, such as rice seedlings, in which this hormone seems to negatively regulate tolerance to salinity^51^. Lastly, there are ecological concerns that need to be addressed before considering large-scale field application. For instance, ACC amendments in soils potentially influence the physiology of surrounding plants and can be utilised by undesirable plants, such as weeds. Hence, we suggest that field trials are conducted including a range of crops cultivated under different conditions to confirm our results and more importantly, the impact of edaphic factors should be assessed. Physical and chemical properties (e.g. texture, nutrient status, pH and water-holding capacities) may affect the availability of ACC and consequently its effect on soil microbial diversity, activity and plant physiology.

Other unknown factors involved in plant-microbe-ACC interactions might have influenced the beneficial magnitude of ACC treatments on the germination and growth of *Arabidopsis*, which led to a relative lower biomass of *Arabidopsis* at 200 µM ACC than at 50 µM ACC treatments in the saline soils. This warrants future studies to investigate in depth by using different soils, ACC concentrations and saline conditions.

## Conclusions

Altogether, this study revealed that microbial community structure and functions are more likely to be positively affected by ACC amendments in soils under salinity stress than non-saline soils. We also provide evidence to suggest that ACC amendments help to improve plant productivity in saline soils. This study opens the door for research aiming to evaluate the commercial feasibility of using ACC to overcome salinity stress for plant production in croplands. Future work is needed to assess this approach in different soil types as soil properties may influence soil responses to ACC. Pending questions also include what are long-term impacts of ACC amendments on soil microbiome and productivity, and if this method can address issues with other stresses (e.g. drought).

## Materials and Methods

### Experimental procedures used to obtain saline soil and ACC treatments

A non-saline soil (silty clay loam) was collected from the A horizon (0–20 cm) of a soil in a garden (27°31′37.0″S 152°59′51.8″E) in Brisbane, Queensland, Australia in March, 2015. The area has a humid subtropical climate with an annual mean minimum of 16.6 °C and mean maximum of 26.6 °C. After collection, the soil was air-dried for 24 hours at room temperature, and then grinded and passed through a 2 mm-sieved to remove plant residues and subsequently homogenized by mixing. This soil had an original electrical conductivity (EC_1:5_) of 1.42 deci-Siemens per meter (dS m^−1^). Other soil physicochemical characteristics including proportions of sand, silt and clay, soil pH and total elements are described in Table S6.

Soil treatments included two levels of salinity (1.42 and 8.13 dS m^−1^) and four levels of ACC (0, 50, 200 and 2,000 µM). To calculate the amount of salt (NaCl) needed for developing the artificially-salinized soils, soil saturation percentage (SP) and water holding capacity (WHC) were firstly determined. Soil EC_1:5_ was then measured to determine the EC of the soil suspension with an electrical conductivity meter (Hach Oakton® Meter, Multi-Parameter, 0.2 kg L^−1^ distilled water). Generally, a soil with an EC of 4 dS m^−1^ (approximately 40 mM NaCl) at 25 °C is considered saline^4^. In this study, we increased the salinity to a level (8 dS m^−1^) that can lead to more than 50% yield loss for most crops worldwide^54^. The amount of sodium chloride required for generating this saline soil was calculated by using the following formula

$${\rm{NaCl}}\,{\rm{required}}\,({\rm{g}}\,{{\rm{kg}}}^{-1}\,{\rm{soil}})=\frac{{\rm{TSS}}\,({\rm{ppm}})\times {\rm{Molecular}}\,{\rm{Weight}}\,{\rm{of}}\,{\rm{NaCl}}\,({\rm{g}})\times {\rm{SP}}}{1000\times 100}$$

where, TSS (ppm) = EC_1:5_ (dS m^−1^) × 10.

The control soil did not receive any salt amendments and was referred to non-saline soil in this study. Soil was dispended to square pots (5 cm^2^ × 12.5 cm deep), and three biological replicates were used per treatment. ACC solutions of four concentrations including 0, 50, 200 and 2,000 µM (Phytotechnology Laboratories^®^, purity ~99.5%) were prepared with autoclaved deionized water. At the time of the treatment application, the calculated amount of ACC solution (in grams) was added in each pot to make 80% soil WHC. Thereafter, to maintain the soil concentration of ACC, the pots were weighed every 3 days and the corresponding amount of water was added to each pot to compensate the weight loss and maintain the ACC concentration throughout the experiment. All the pots were incubated at 25 °C in a growth chamber (Percival Scientific, Boone, IA, USA). Soil sampling was carried out 14 days after the ACC amendments and part of the soil samples were stored in 50 mL sterile Falcon tubes at 4 °C until the completion of soil analysis for metabolic and enzyme activities (~1 week). A small fraction of each soil sample (~20 g) was preserved at −20 °C for genomic DNA extraction.

### Soil DNA extraction

Total genomic DNA of each soil sample was extracted from 0.25 g soil using the MO BIO’s PowerSoil DNA Isolation Kit (Qiagen). DNA quality was examined with a Nanodrop™ (Thermo Scientific™) spectrophotometer and concentrations were measured using the Qubit™ fluorometer with Quant-iT dsDNA HS Assay Kits (Invitrogen). All soil DNA samples were then normalized to 5 ng µL^−1^.

### Microbiome responses to treatments

For profiling soil bacterial and archaeal communities, eubacterial and archaeal 16S rRNA genes were amplified by PCR using the primer pair 926F (5′-AAA CTY AAA KGA ATT GRC GG-3′)^55^ and 1392R (3′-ACG GGC GGT GWG TRC-5′)^56^. PCRs were performed in duplicate on each DNA sample. There were 14.75 µL of ultra-pure water, 5 µL of 5 × Phire buffer (Thermo Scientific), 1.25 µL of dNTPs (10 µM), 1.25 µL of a 10 µM 926F, 1.25 µL of a 10 µM 1492R, 0.5 µL of Phire® hot-start II (Thermo Scientific) and 1 µL (5 ng) of DNA template in each PCR reaction. Thermal conditions used for the above amplification were 30 s at 98 °C for initial denaturation, 30 cycles of 15 s at 98 °C, 30 s at 56 °C for annealing and 45 s at 72 °C; followed by 7 min at 72 °C for final extension. Purification of PCR products were conducted using Agencourt AMPure XP beads (Beckman Coulter, Inc.). Purified amplicons were dual indexed using the Nextera XT Index Kit (Illumina) according to the manufacturer’s instructions. Indexed amplicons were first purified using Agencourt AMPure XP beads and then quantified using the Qubit™ fluorometer with Quant-iT dsDNA HS Assay Kits (Invitrogen). The pooled amplicons composed of equal concentrations of each sample were sequenced on an Illumina MiSeq using 25% PhiX Control v3 (Illumina) and a MiSeq Reagent Kit v3 (600 cycle; Illumina). Amplicon sequencing for soil fungal communities was performed by the Australian Centre for Ecogenomics (ACE) using the Illumina MiSeq platform. The internal transcribed spacer (ITS2) region of the ribosomal RNA was amplified using the primer pair gITS7 (5′-GTG ART CAT CGA RTC TTTG-3′)^57^ and ITS4 (5′-TCC TCC GCT TAT TGA TAT GC-3′)^58^.

### Bioinformatics analysis

#### 16S rRNA sequencing

Raw sequences from 16S rRNA sequencing were processed as previously described (Liu *et al*., 2016). Briefly, primer sequences were removed from each fastq file using the QIIME v1.9.1 software package. Sequences of each file was then quality filtered and dereplicated using the QIIME script multiple_split_libraries.py with the homopolymer filter deactivated^59^. The forward reads from each sample were concatenated into a single file. Chimeras were checked against the GreenGenes database using UCHIME ver. 3.0.617^60^ and homopolymer errors were corrected using Acacia^61^. Sequences were then subjected to the following procedures using QIIME: (i) sequences were clustered at 97% similarity using UCLUST, (ii) GreenGenes (version 2013/05) taxonomy was assigned to the cluster representatives using BLAST, and (iii) tables with the abundance of different operational taxonomic unit (OTUs) and their taxonomic assignments in each sample were generated. The number of reads was rarefied to 6,000 per sample by re-sampling the OTU table. The mean number of observed (Sobs) and predicted (Chao1) OTUs, Simpson’s diversity index values, and Shannon’s diversity index values corresponding to 6,000 sequences were calculated using QIIME.

#### ITS sequencing analysis

All fastq files were processed with fastqc to check the completion status of the sequencing. The first 19 bases of all fastq files were trimmed to remove primer sequences and further trimmed to remove sequences of poor quality using the software Trimmomatic^62^. All reads were then hard trimmed to 250 bases and those sequences with lengths less than 250 bases were excluded. For read clustering and taxonomy assignment, fasta files were processed by the script of pick_open_reference_otus.py in QIIME with default parameters (97% similarity) and taxonomy assignment and alignment features suppressed. The OTU table obtained was filtered to remove any OTU with an abundance of less than 0.05%. Representative OTU sequences were then BLASTed against the reference database UNITE singleton included release 04/07/2014. All samples had their reads rarefied to 29,000. Lifestyle status for fungal OTUs was predicated using the FUNGuild database by using an online version of the Guilds bioinformatics tool (http://funguild.org)^63^. For analyses, five guilds were used, namely plant pathogen-soil saprotroph-wood saprotroph, plant pathogen, undermined saprotroph, animal pathogen and undetermined.

### Analysis of soil microbial functions

Total soil microbial enzyme activity (MEA) and substrate-induced respiration were measured using the fluorescein diacetate hydrolysis (FDA) assay and Microresp™, respectively, according to the previously described protocols^64^. For the MEA measurements, 2 g of fresh soil were incubated in 15 mL potassium phosphate buffer (60 mM, pH 7.6) in a sterile Falcon tube. The reaction was started by adding 200 μL of a 1,000 μg mL^−1^ FDA solution into the tube as the substrate and shaking at 150 rpm and 30 °C for 1 h. To stop the reaction, 950 μL of this mixture was transferred to a new 2 mL tube that contained the same volume of 2:1 (v:v) chloroform/methanol. The obtained soil suspension was subsequently centrifuged for 3 min at 12,000 g and 250 μL of the supernatant was dispended into a 96-well plate. The plate was then read at 450 nm in a microtiter plate reader (BMG Lab, Ortenberg, Germany).

The soil community level physiological profiles (CLPP) was measured using a multi-substrate induced respiration (SIR) approach with the MicroResp™ system (James Hutton Institute, Invergowrie, Scotland, UK)^65^. Fifteen pre-dispensed C sources including carboxylic acids (citric acid, L-malic acid, methyl pyruvate, oxalic acid), amino acids (alanine, proline), carbohydrates (arabinose, D-glucose, D + cellubiose, sucrose, maltose, mannitol, β-d-fructose) and two polymers (Tween 20, Tween 40) were used as C substrates. Milli-Q deionized water was used as a control, which accounts for basal level soil respirations. Approximately 0.40 g soil was transferred into each well of a deep-well plate and the soil moisture was adjusted to 30% with milli-Q water. The soil plate was then incubated in a sealed plastic box containing self-indicating soda lime at 25 °C for 3 days. Each C substrate was amended to deep wells in triplicates to provide 7.5/30 mg C g^−1^ soil water according to Campbell *et al*. (2003). The assembled MicroResp™ system was incubated at 25 °C for 6 h and the color development was measured with a microplate spectrophotometer (BioTck, Winooski VT, USA) at 570 nm. The CO_2_ production (μg CO_2_-C g^−1^ h^−1^) was calculated based on the difference between the absorbance at 6 h and 0 h.

### Potential responses of functional microbiomes involved in N and C cycling and ACC deaminase production

The primers used for measuring copy numbers of *amoA* (bacterial ammonia monooxygenase subunit A), *nifH* (nitrogenase), arch-*amoA* (archaeal ammonia monooxygenase subunit A), *narG* (nitrate reductase), *nirK* (nitrite reductase), *chiA* (chitinaseA) and *acdS* (ACC deaminase) genes are listed in Table S2. All qPCR reactions contained 1.5 µL 0.3 µM of each forward and reverse primer, 5 µL 2 × Faststart SYBR green mix (Roche Diagnostics Ltd), 1 µL DNA template (2.5 ng) and 2.5 µL nuclease free water. Cycling conditions for *amoA*, *nifH*, arch-*amoA*, *narG*, *nirK* and *chiA* included an initial step at 98 °C for 10 min, followed by 45 cycles of 98 °C for 30 s, annealing for 45 s, and elongation at 72 °C for 45 s. Cycling conditions for *acdS* gene included an initial step at 95 °C for 10 min, followed by 50 cycles of 95 °C for 15 s, annealing 10 s, and elongation at 72 °C 30 s. Dissociation curves were generated for all primer sets by adding the cycle, 95 °C for 10 s, 65 °C for 60 s and 97 °C for 1 s at reduced ramping rate of 0.2 °C s^−1^ to check for unspecific amplification. PCR amplification data were analyzed using Light Cycler® 96 software. The optimal annealing temperature for each primer pair was tested by using a temperature gradient (Table S7). The specificity of qPCR amplification was firstly confirmed by a single melting peak and secondly by a single band on a 1.5% agarose gel. PCR products were then excised from the gel and then purified using the Wizard® SV Gel and PCR Clean-Up System (Promega). Purified PCR products were sent to the Australian Genome Research Facility Ltd (AGRF) for Sanger sequencing. Distance-based clusterings generated by pairwise alignments with the query sequence in BLAST indicated that the excised bands corresponded to the targeted genes. Ten-fold sequential dilutions of purified amplification products were used to generate standard curves with the abovementioned conditions. Gene copy numbers in soil samples were quantified by comparing the Ct values gained by qPCR against the corresponding standard curve. The final gene copies for each sample were normalized to per gram fresh soil.

### Effects of ACC and salinity treatments on soil productivity

Soil productivity after ACC and salinity treatments was assessed using the model plant *A*. *thaliana* (Col-0). Plants were cultivated in seedling punnet black trays filled with soils that received the same treatments as previously described. Four biological replicates were used per treatment and each biological replicate contained 60 seeds. Plant trays were kept at 4 °C for two days for vernalization and were then transferred to a growth chamber (Percival Scientific, Boone, IA, USA) with a light intensity of 150 µmol m^−2^ s^−1^ at 24 °C. The positions of the two trays were changed daily throughout experiments to minimize environmental variability within the growth chamber. Plants (roots and shoots combined) were harvested after two weeks and rinsed with distilled water to remove soils, air-dried and weighed for fresh biomass.

### Statistical analysis

General patterns on the effects of ACC and salinity treatments on univariate response variables such as soil MEA, alpha diversity metrics, C substrate utilization and plant biomass production were analyzed using analysis of variance (ANOVA) with LSD at 95% confidence. The effects of ACC and salinity on multivariate responses, such as soil utilization of C substrates, OTU abundances and unifrac distances of microbial communities were investigated using permutational multivariate analysis of variance (PERMANOVA). Differences in particular microbial taxa between samples were analyzed by STAMP (2.1.3) using the ANOVA of Tukey-Kramer test with Bonferroni correction for multiple tests^66^. Rank transformation was performed for ACC concentrations prior to the abovementioned statistical analysis. Further, phylogenetic molecular ecological networks for salinity treatments were analyzed using the R package SPIEC-EASI^67^. Graphs were created by R (3.0.2), GraphPad or Calypso 8.84 (Zakrzewski *et al*., 2017), and were then edited accordingly using Inkscape (0.92.1).

Finally, we used structural equation modelling (SEM)^68^ to evaluate the direct and indirect effects of salinity and ACC on soil function and plant biomass via changes in microbial community. Microbial community composition was included in our model as the three axes of an NMDS conducted on bacterial and fungal composition data at the OTU level (stress = 0.09 and =0.04, respectively)^69^. Salinity was included as a categorical variable with two levels: 1 (salinity) and 0 (control). Selected soil functions (those strongly correlating with plant biomass) were included in our model. We tested the overall goodness of fit of our model by using Chi-square test (χ2; the model has a good fit when 0 ≤ χ/d.o.f ≤ 2 and 0.05 ≤ P ≤ 1.00) and the root mean square error of approximation (RMSEA; the model has a good fit when *RMSEA* 0 ≤ *RMSEA* ≤ 0.05 and 0.10 ≤ *P* ≤ 1.00)^70^. Because some variables did not follow the normal distribution, we confirmed the fit of the model using the Bollen-Stine bootstrap test (the model has a good fit when 0.10 < bootstrap P $\leqslant$ 1.00). SEM analysis was conducted with the software AMOS 20 (IBM SPSS Inc, Chicago, IL, USA).

## Supplementary information

Supplementary materials of this paper

## Data Availability

All datasets generated and/or analysed during the current study are available in the Figshare (https://figshare.com/s/0f9f5c5829fb2213e472). Soil information, bacterial and fungal taxonomy in details are included in this published article and its supplementary information files. The 16S rRNA amplicon sequences associated with this study have been deposited in the NCBI SRA accession: PRJNA368996; ITS amplicon sequences were deposited in the NCBI SRA with accession ID as PRJNA396974.
