# Supplementary material for: Soil amendments with ethylene precursor alleviate negative impacts of salinity on soil microbial properties and productivity
Source: Sci Rep. 2019 May 3;9:6892. doi: 10.1038/s41598-019-43305-4 (PMC6499801; doi:10.1038/s41598-019-43305-4)
Supplement: Supplementary file 1 — Supplementary materials of this paper [file 41598_2019_43305_MOESM1_ESM.pdf]

Supplementary information for **"Soil amendments with ethylene precursor alleviate negative impacts of salinity on soil microbial properties and productivity"**

Hongwei Liu[1,2#*], Muhammad Yahya Khan[3#], Lilia C. Carvalhais[4], Manuel Delgado-Baquerizo[5], Lijuan Yan[6], Mark Crawford[7], Paul G. Dennis[8], Brajesh Singh[2], Peer M. Schenk[1]

[1]Plant-Microbe Interactions Laboratory, School of Agriculture and Food Sciences, The University of Queensland, Brisbane, Queensland 4072, Australia; [2]Hawkesbury Institute for the Environment, Western Sydney University, Penrith, New South Wales 2751, Australia; [3]Institute of Soil and Environmental Science, The University of Agriculture Faisalabad, Faisalabad 38000, Pakistan; [4]Centre for Horticultural science, Queensland Alliance for Agriculture and Food Innovation, The University of Queensland, Brisbane, Queensland, Australia; [5]Cooperative Institute for Research in Environmental Sciences, University of Colorado, Boulder, CO 80309,USA; [6]Institute of Biodiversity, Friedrich Schiller University Jena 07749, Germany; [7]Department of Natural Resources and Mines, Toowoomba, QLD, Australia; [8]School of Earth and Environmental Sciences, The University of Queensland, Brisbane, QLD 4072, Australia.

[#]Equal contribution

*Correspondence: h.liu2@westernsydney.edu.au

Table of contents:

Table S1 Effects of ACC treatments on bacterial/archaeal taxa in saline soils............................3

Table S2 Effects of ACC treatments on bacterial/archaeal taxa in non-saline soils....................4

Table S3 Phylogenetic molecular ecological network analysis..5

Table S4 Effects of salinity treatments on soil fungal taxa...6

Table S5 Pearson correlation between the axes from NMDSs and single taxa............................7

Table S6 Soil physicochemical properties..8

Table S7 Primers used for measuring genes involved in C and N cycling..................................9

Fig.S1 Heatmap summarising variations in bacterial and archaeal taxa....................................10

Fig.S2 Effects of ACC and salinity treatments on soil microbial phyla.....................................11

Fig.S3 Changes in network structure of soil prokaryotic communities.....................................12

Fig.S4 Soil microbial diversity...13

Fig.S5 ACC effects on soil carbon substrate utilization..14

Fig.S6 SEM analysis..15

References...16

Table S1. Soil bacterial populations (operational taxonomic units (OTUs)) that were influenced by ACC treatments in the saline soils.

OTU_ID	p-values	P-values (corrected)	0: rel. freq. (%)	50: rel. freq. (%)	200: rel. freq. (%)	2000: rel. freq. (%)	Taxonomy
3	0.034	0.376	2.74±0.73	2.02±0.14	1.85±0.04	1.27±0.24	p_Acidobacteria; c_Acidobacteria; o_Acidobacteriales; f_unclassified; g_unclassified
10	0.014	0.150	0.17±0.08	0.43±0.22	0.61±0.05	1.26±0.44	p_Actinobacteria; c_Actinobacteria; o_Actinomycetales; f_*Microbacteriaceae* ; g_*Microbacterium*
36	0.008	0.093	1.17±0.22	0.39±0.04	0.64±0.15	0.57±0.20	p_SPAM; c_0319-6G9; o_unclassified; f_unclassified; g_unclassified
27	0.028	0.311	0.47±0.08	0.27±0.04	0.30±0.05	0.39±0.04	p_Proteobacteria; c_Betaproteobacteria; o_unclassified; f_unclassified; g_unclassified
OTU_56069	0.012	0.128	0.63±0.05	0.54±0.05	0.37±0.07	0.39±0.09	p_Acidobacteria;c_Acidobacteria;o_Acidobacteriales;f_unclassified;g_unclassified
OTU_63594	0.031	0.341	0.28±0.06	0.41±0.11	0.39±0.07	0.58±0.06	p_Actinobacteria;c_Actinobacteria;o_Solirubrobacterales;f_*Solirubrobacteraceae* ;g_unclassifed
OTU_76249	0.008	0.083	0.15±0.04	0.28±0.07	0.34±0.08	0.46±0.05	p_Actinobacteria;c_Actinobacteria;o_Actinomycetales;f_*Microbacteriaceae* ;g_*Agromyces*
OTU_47099	0.023	0.248	0.06±0.03	0.22±0.05	0.20±0.06	0.38±0.13	p_Actinobacteria;c_Actinobacteria;o_Actinomycetales;f_*Microbacteriaceae* ;g_*Microbacterium*
OTU_72418	0.049	0.541	0.21±0.02	0.25±0.05	0.33±0.04	0.37±0.08	p_Actinobacteria;c_Actinobacteria;o_MC47;f_unclassified;g_unclassified
OTU_62989	0.029	0.321	0.30±0.02	0.35±0.03	0.34±0.04	0.25±0.02	p_Acidobacteria;c_Acidobacteria;o_Acidobacteriales;f_unclassified;g_unclassified
OTU_55567	0.004	0.044	0.06±0.02	0.14±0.08	0.21±0.05	0.32±0.02	p_Actinobacteria;c_Actinobacteria;o_Actinomycetales;f_*Microbacteriaceae* ;g_*Microbacterium*

The OTUs shown differed between the control and ACC treatments in the saline soils using ANOVA with Tukey-Kramer tests for post-hoc comparisons of means as implemented in STAMP. The values shown in ACC (level 0, 50, 200, 2000 μM) columns are the mean relative abundances within each treatment ±SDs. Shown are OTUs present at ≥0.3% mean relative abundance in different ACC treatments.

Table S2. Soil prokaryotic populations (operational taxonomic units (OTUs)) that were influenced by ACC treatments in the non-saline soils.

OTU_ID	p-values	p-values (corrected)	0: rel. freq. (%)	200: rel. freq. (%)	2000: rel. freq. (%)	50: rel. freq. (%)	Taxonomy
1	0.028	0.167	1.00±0.15	1.64±0.02	1.61±0.35	1.00±0.23	k_Archaea; p_Crenarchaeota; c_Thaumarchaeota; o_Nitrososphaerales; f_*Nitrososphaeraceae*; g_*Nitrososphaera*
33	0.005	0.030	0.42±0.06	0.70±0.11	0.51±0.08	0.32±0.01	k_Bacteria; p_Proteobacteria; c_Gammaproteobacteria; o_Chromatiales; f_*Sinobacteraceae*; g_unclassified
OTU_60523	0.000	0.002	0.47±0.04	0.28±0.01	0.26±0.04	0.24±0.03	k_Bacteria;p_Acidobacteria;c_Acidobacteria;o_Acidobacteriales;f_unclassified;g_unclassified
OTU_8606	0.048	0.287	0.10±0.03	0.24±0.09	0.38±0.01	0.25±0.12	k_Bacteria;p_Actinobacteria;c_Actinobacteria;o_Actinomycetales;f_*Microbacteriaceae*;g_*Microbacterium*
OTU_63984	0.021	0.126	0.31±0.04	0.19±0.06	0.14±0.06	0.12±0.03	k_Bacteria;p_Nitrospirae;c_Nitrospira;o_Nitrospirales;f_*Nitrospiraceae*;g_*Nitrospira*
OTU_32822	0.010	0.063	0.30±0.04	0.15±0.04	0.17±0.02	0.10±0.06	k_Bacteria;p_Acidobacteria;c_Acidobacteria;o_Acidobacteriales;f_unclassified;g_unclassified

The OTUs shown differed between the control and ACC treatments in the non-saline soils using ANOVA with Tukey-Kramer tests for post-hoc comparisons of means as implemented in STAMP. The values shown in ACC (level 0, 50, 200, 2000 µM) columns are the mean relative abundances within each treatment ±SDs. Shown are OTUs present at ≥0.3% mean relative abundance in different ACC treatments.

Table S3. Changes in bacterial/fungal communities in response to salinity treatments using phylogenetic molecular ecological networks (pMENs).

Parameters	Bacteria		Fungi	
	Control	Saline	Control	Saline
Clustering coefficient	0.038	0.01	0	0.023
Connected component	17	16	25	18
Network diameter	17	12	4	14
Short paths	4110 (36%)	2214(33%)	160 (3%)	3330 (31%)
Average number of neighbours	1.83	1.71	1.31	1.81
Number of nodes	106	82	70	103
Network heterogeneity	0.584	0.545	0.473	0.578

Table S4. Soil fungal operational taxonomic units (OTUs) that were influenced by salinity treatments.

OTU_ID	P value	P value corrected	Salinity: rel. freq. (%)	non-salinity: rel. freq. (%)	Taxonomy
OTU_8	0.0000	0.0002	4.00±1.24	1.83±0.39	p_Ascomycota;c_Sordariomycetes;o_Hypocreales;f_*Nectriaceae* ;g_*Haematonectria*
OTU_9	0.0002	0.0027	3.16±1.18	1.51±0.36	p_Ascomycota;c_Sordariomycetes;o_Hypocreales;f_*Nectriaceae* ;g_*Fusarium*
OTU_3	0.0002	0.0030	10.16±1.90	7.16±1.25	p_Basidiomycota;c_Tremellomycetes;o_Trichosporonales;f_*Trichosporonaceae* ;g_*Trichosporon*
OTU_4	0.0003	0.0035	4.81±1.92	2.20±0.57	p_Ascomycota;c_Sordariomycetes;o_Hypocreales;f_*Nectriaceae* ;g_*Fusarium*
OTU_27	0.0006	0.0076	0.77±0.38	0.29±0.12	p_Ascomycota;c_Sordariomycetes;o_Hypocreales;f_*Nectriaceae* ;g_*Fusarium*
OTU_1	0.0009	0.0123	9.88±10.87	26.27±9.22	p_Ascomycota;c_Pezizomycetes;o_Pezizales;f_*Pezizaceae* ;g_unidentified
OTU_21	0.0051	0.0660	0.57±0.22	0.96±0.36	p_unidentified;c_unidentified;o_unidentified;f_unidentified;g_unidentified
OTU_2	0.0076	0.0982	21.59±7.61	14.09±3.69	p_Basidiomycota;c_Tremellomycetes;o_Trichosporonales;f_*Trichosporonaceae* ;g_*Trichosporon*
OTU_25	0.0150	0.1950	0.70±0.27	0.41±0.25	p_Ascomycota;c_unidentified;o_unidentified;f_unidentified;g_unidentified
OTU_12	0.0300	0.3900	1.38±0.39	1.91±0.64	p_Ascomycota;c_Sordariomycetes;o_Sordariales;f_*Chaetomiaceae* ;g_*Thielavia*
OTU_13	0.0410	0.5330	1.34±0.34	1.64±0.33	p_unidentified;c_unidentified;o_unidentified;f_unidentified;g_unidentified
OTU_30	0.0410	0.5330	0.75±0.76	0.24±0.10	p_Ascomycota;c_Sordariomycetes;o_Hypocreales;f_*Nectriaceae* ;g_*Fusarium*
OTU_26	0.0450	0.5850	0.35±0.29	0.72±0.48	p_Zygomycota;c_Incertae_sedis;o_Mortierellales;f_*Mortierellaceae* ;g_*Mortierella*

The OTUs shown differed between the saline and non-saline conditions using ANOVA with Tukey-Kramer tests for post-hoc comparisons of means as implemented in STAMP. The values shown in saline/non-saline columns are the mean relative abundances within each treatment ±SDs. Shown are OTUs present at ≥0.5% mean relative abundance in different salinity treatments.

Table S5. The correlation between the axes from NMDSs and single taxa tested using the linear correlation test (Pearson).

Bacterial community (OTU>0.05% in relative abundance)

BACT_1			BACT_2		
OTU_ID	r	Sig.	OTU_ID	r	Sig.
3	0.66	***	1	0.8	***
4	0.64	***	2	0.64	***
5	0.53	**	9	-0.44	*
6	0.64	***	14	-0.47	*
8	-0.7	***	17	0.57	**
9	-0.74	***	18	0.67	***
10	-0.69	***	25	-0.44	*
11	-0.88	***	28	-0.51	*
12	-0.82	***	33	0.41	*
13	-0.68	***			
14	-0.77	***			
15	-0.88	***			
16	-0.69	***			
17	0.51	*			
19	-0.54	**			
20	-0.55	**			
21	0.48	*			
22	0.67	***			
24	0.48	*			
26	0.57	**			
27	0.53	**			
29	0.61	***			
30	0.58	**			
31	0.77	***			
32	0.82	***			
36	0.81	***			
37	0.78	***			

Fungal community (Top 10 OTUs)

FUNG_1			FUNG_2		
OTU_ID	r	Sig.	OTU_ID	r	Sig.
38	-0.95	***	40	-0.42	*
39	0.82	***	43	-0.54	**
40	0.8	***	45	0.68	***
41	0.9	***	46	0.63	**
43	0.76	***	47	-0.44	*
44	0.7	***			
47	0.42	*			

Table S6. Soil physicochemical properties.

Soil parameter	Value
Sand (%)	12
Silt (%)	50
Clay (%)	38
pH	7.8
EC (dS m^{-1})	1.42
Total carbon content (Wt%)	1.13
Total nitrogen content (Wt%)	0.04
Total Al concentration (Wt%)	4.86
Total As concentration (mg kg^{-1})	0.84
Total Ba concentration (mg kg^{-1})	251
Total Ca concentration (Wt%)	1.3
Total Cd concentration (mg kg^{-1})	0.35
Total Co concentration (mg kg^{-1})	2.47
Total Cr concentration (mg kg^{-1})	78
Total Cu concentration (mg kg^{-1})	52
Total Fe concentration (Wt%)	5.63
Total K concentration (Wt%)	3.6
Total Mg concentration (Wt%)	0.21
Total Mn concentration (mg kg^{-1})	1295
Total Mo concentration (mg kg^{-1})	1.44
Total Na concentration (Wt%)	2.91
Total Ni concentration (mg kg^{-1})	59
Total P concentration (Wt%)	0.31
Total Pb concentration (mg kg^{-1})	0.18
Total S concentration (mg kg^{-1})	1836
Total Se concentration (mg kg^{-1})	0.33
Total Si concentration (Wt%)	32
Total Sr concentration (mg kg^{-1})	190
Total Zn concentration (mg kg^{-1})	315

Table S7. Primer sequences used for real-time PCR assays designed for the determination of copy numbers of nitrogen and carbon cycling genes in soil.

Genes	Forward sequence	Reverse sequence	Amplicon (bp)	Tm[A] (°C)	Reference
Chitinase A	CGTCGACATCGACTGGGARTDBCC	ACGCCGGTCCAGCCNCKNCCRTA	400	63	(Yergeau et al., 2007)
arch-amoA	TTCTTCTTTGTTGCCCAGTA	CTGAYTGGGCYTGGACATC	256	63	(Wuchter et al., 2006)
nifH	AAAGGYGGWATCGGYAARTCCACCAC	TTGTTSGCSGCRTACATSGCCATCAT	459	60	(Rösch et al., 2002)
amoA	GGGGTTTCTACTGGTGGT	CCCCTCKGSAAAGCCTTCTTC	491	56	(Rotthauwe et al., 1997)
nosZ	CGYTGTTCMTCGACAGCCAG	CATGTGCAGNGCRTGGCAGAA	700	64	(Rösch et al., 2002)
narG	TAYGTSGGSCARGARAA	TTYTCRTACCABGTBGC	650	59	(Philippot et al., 2002)
acdS	GGCAACAAGMYSCGCAAGCT	CTGCACSAGSACGCACTTCA	133	67	(Bouffaud et al., 2018)

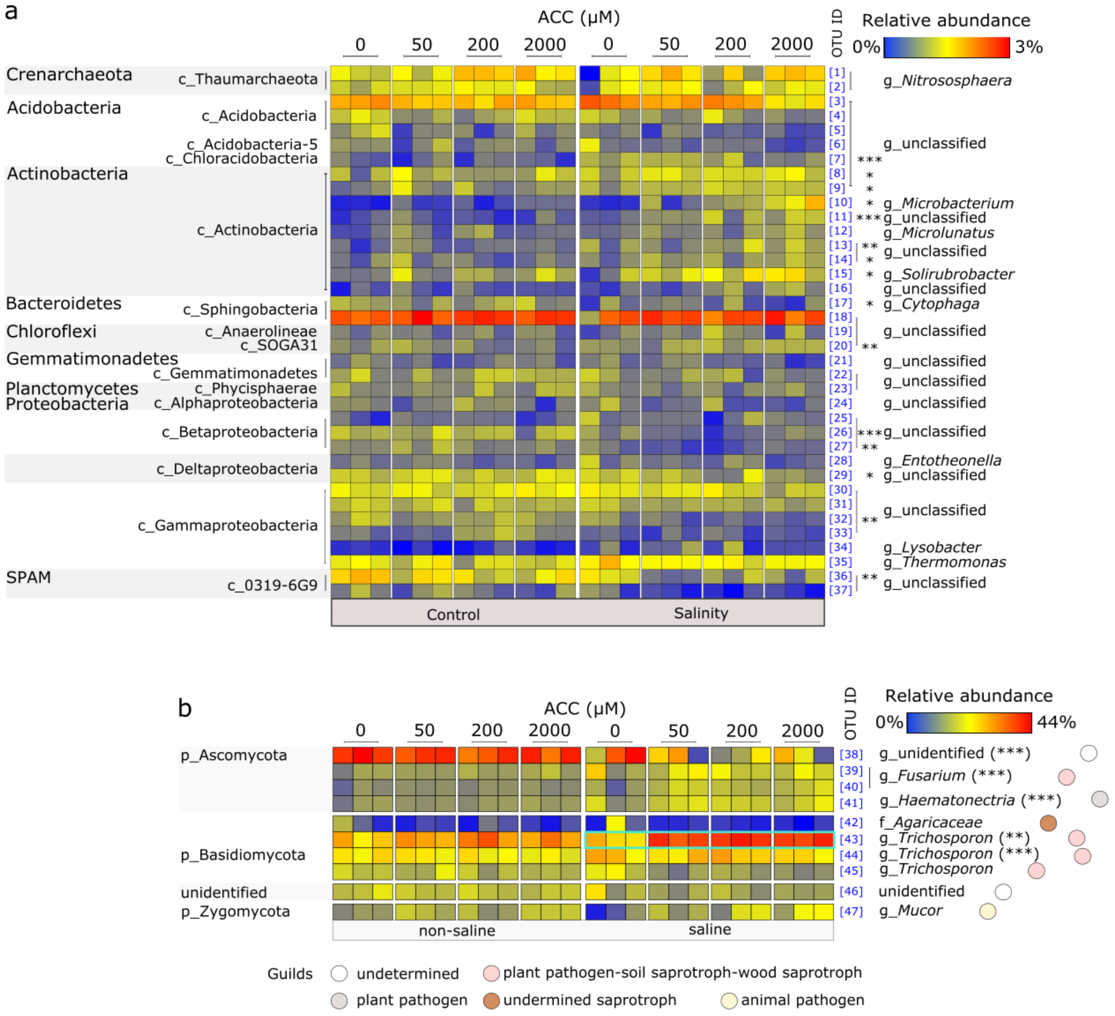

Fig.S1 Heatmap summarizing variations in the microbial taxa based on data obtained from 16S rRNA and ITS amplicon sequencing (shown are taxa ⩾0.5% in relative abundance). Each Operational Taxonomic Unit (OTU) has a unique bracket that is consistent with those shown in other figures and tables. Stars after each OTU ID represent significant differences between saline and non-saline conditions (* P<0.05, ** P<0.01, *** P<0.001, ANOVA, Tukey-Kramer test). The green box inserted in heatmap (**b**) highlights the differences of OTU [42] between control and ACC treatments in the saline soil.

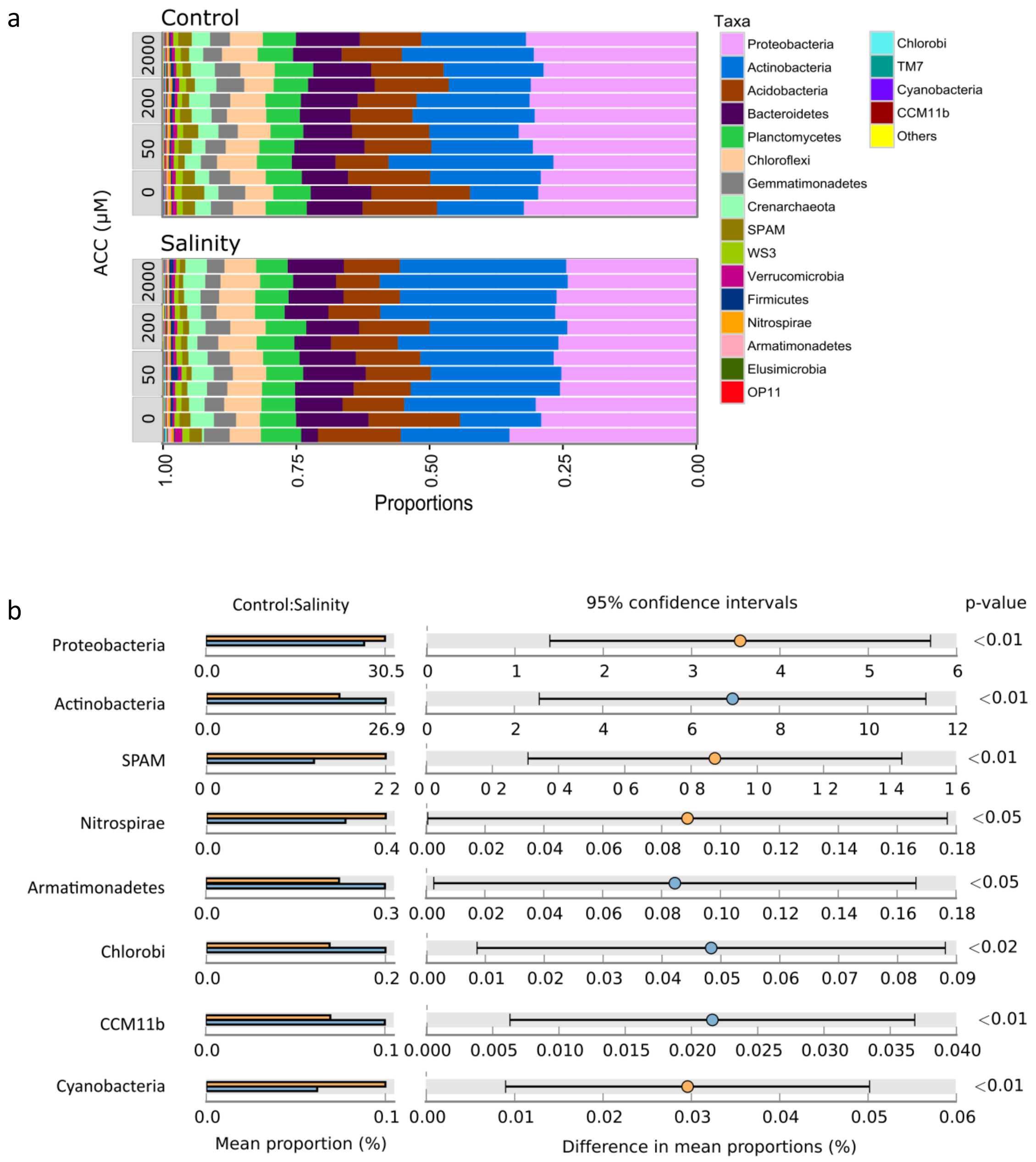

Fig.S2 Barplot summarizing top 20 bacterial/archaeal phyla in the silty clay loam (a) and those bacterial phyla responding to salinity treatments in relative abundances (b). The yellow and blue dots embedded in error bars distinguish decreases and increases in relative abundance of bacterial phyla after salinity treatments, respectively.

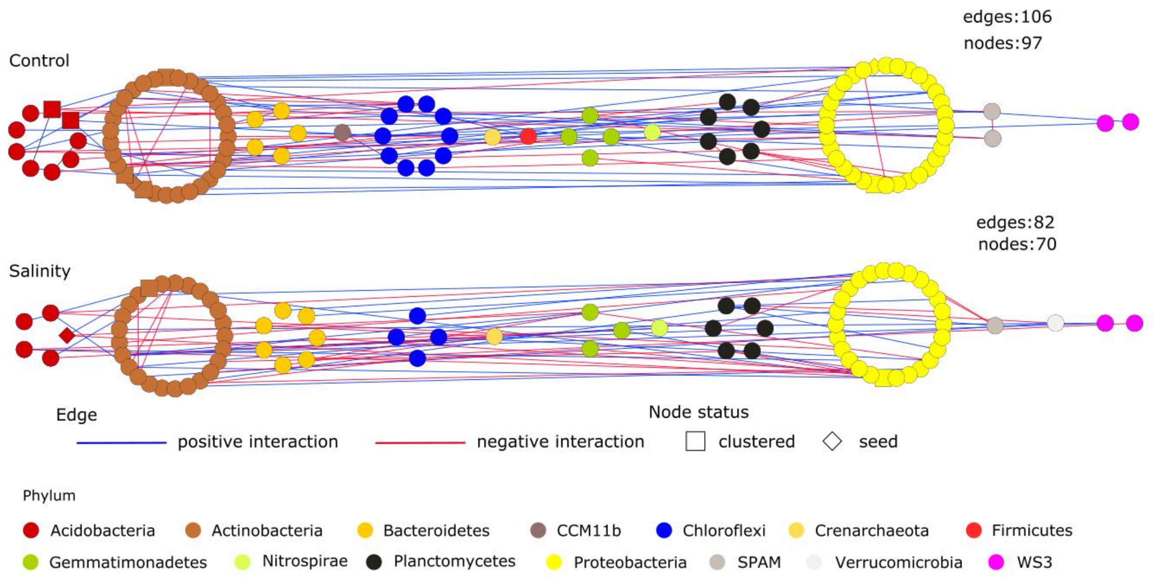

Fig.S3 Changes in soil bacterial/archaeal network structure in response to salinity treatments.

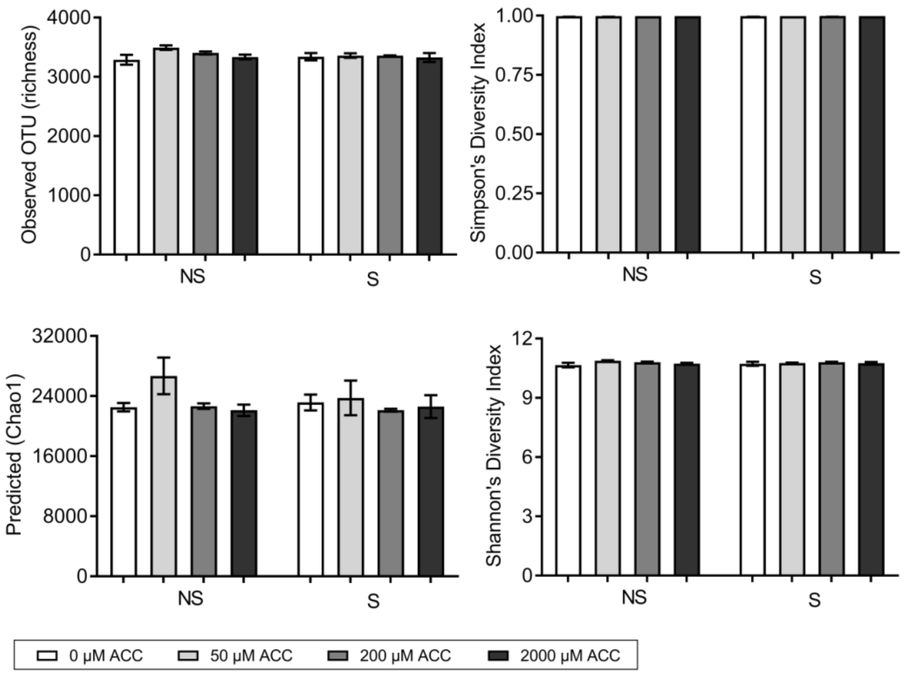

Fig.S4 The effect of ACC and salinity treatments on the observed species, predicted richness and evenness (Simpson's Diversity Index) of bacterial communities associated with soil environments. All values were based on 6,000 rarefied sequences per sample. Error bars denote SEs (n=3).

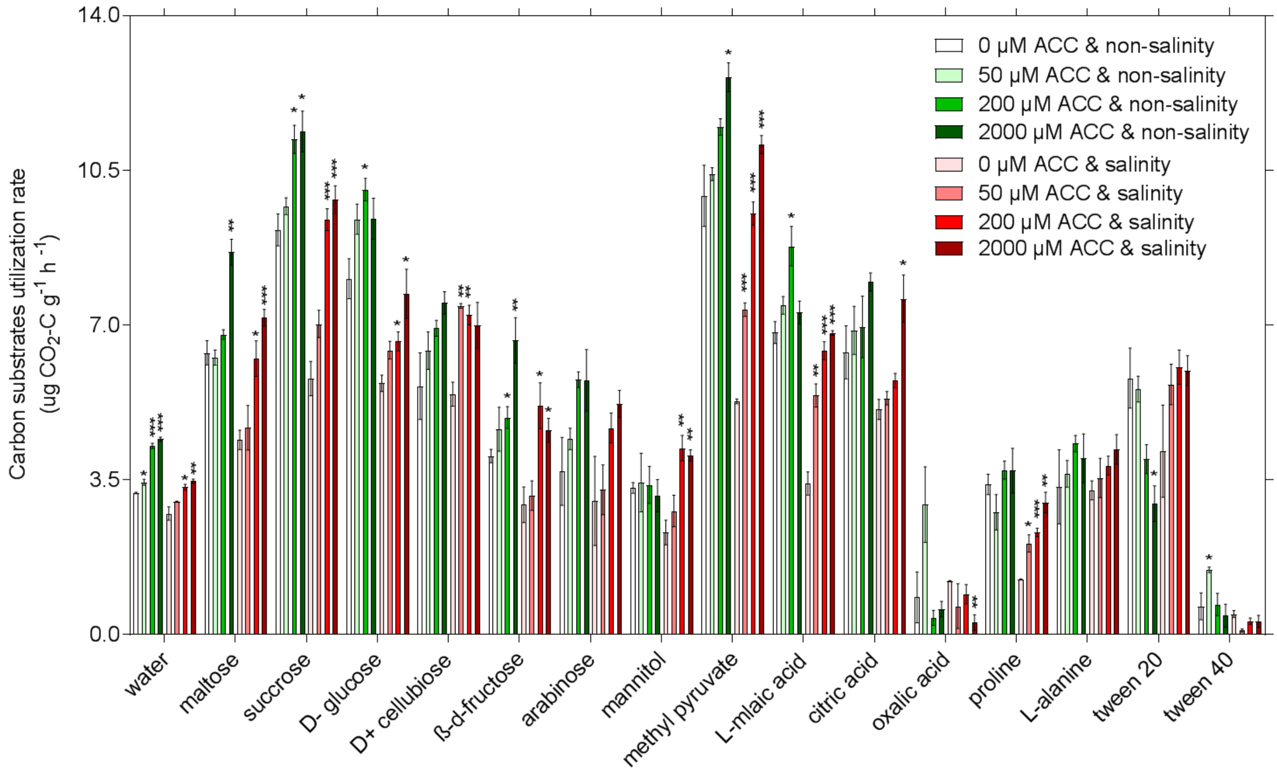

Fig.S5 The effects of ACC amendments on the utilization of 15 carbon substrates in the non-saline and saline soils. Stars above columns represent significant differences between ACC treatments and control (0 μM ACC) (*P*<0.05 *, *P*<0.01 **, *P*<0.001 ***, ANOVA, Least Significant Difference (LSD)). Error bars denote SDs (n=3).

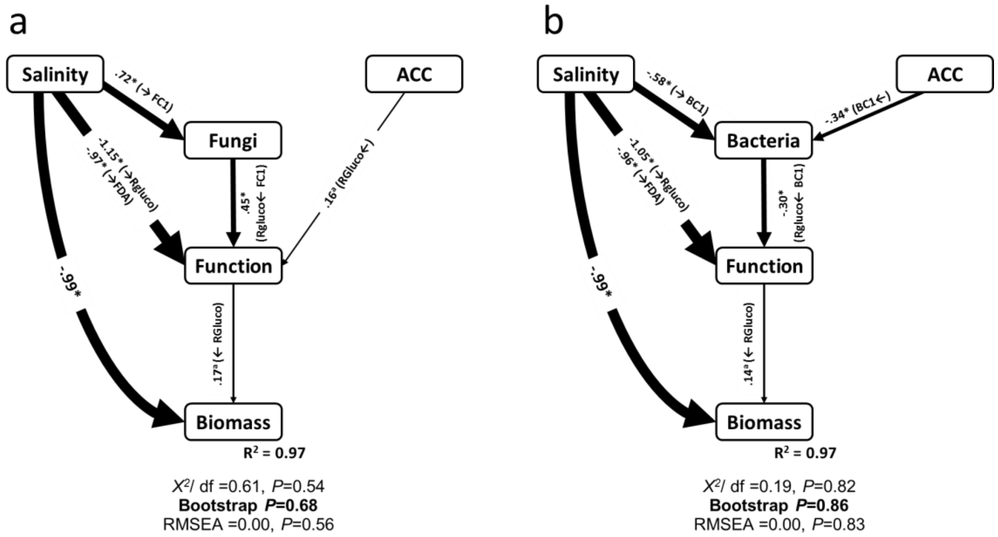

Figure S6. Structural equation models accounting for the direct and indirect effects of salinity and ACC on soil function and plant biomass via changes in microbial community composition (a, b). Numbers adjacent to arrows are path coefficients (P<0.05*, P<0.1a), and indicative of the effect size of the relationship. The thickness of the arrow indicates the strength of relationship. R^2, the proportion of variance explained. Biomass: plant biomass. Function: soil microbial function.

References

Bouffaud, ML., Renoud, S., Dubost, A., Moënne-Loccoz, Y., Muller, D. 2018. 1-Aminocyclopropane-1-carboxylate deaminase producers associated to maize and other Poaceae species. Microbiome 6, 114.

Philippot, L., Piutti, S., Martin-Laurent, F., Hallet, S., Germon, J.C., 2002. Molecular analysis of the nitrate-reducing community from unplanted and maize-planted soils. *Appl Environ Microbiol* 68, 6121-6128.

Rösch, C., Mergel, A., Bothe, H., 2002. Biodiversity of denitrifying and dinitrogen-fixing bacteria in an acid forest soil. *Appl Environ Microbiol* 68, 3818-3829.

Rotthauwe, JH., Witzel, KP., Liesack, W., 1997. The ammonia monooxygenase structural gene amoA as a functional marker: molecular fine-scale analysis of natural ammonia-oxidizing populations. *Appl Environ Microbiol* 63, 4704-4712.

Wuchter, C., Abbas, B., Coolen, M.J., Herfort, L., van Bleijswijk, J., Timmers, P., Strous, M., Teira, E., Herndl, G.J., Middelburg, J.J., 2006. Archaeal nitrification in the ocean. *PNAS* 103, 12317-12322.

Yergeau, E., Kang, S., He, Z., Zhou, J., Kowalchuk, G.A., 2007. Functional microarray analysis of nitrogen and carbon cycling genes across an Antarctic latitudinal transect. *ISME J* 1, 163-179.
